# Transitions in women’s experience of physical domestic violence during 2001–2020 and related risk and protective factors: the MINIMat longitudinal cohort study in rural Bangladesh

**DOI:** 10.1136/bmjgh-2024-018458

**Published:** 2025-12-23

**Authors:** Ruchira Tabassum Naved, Jannatul Ferdous Antu, Mahfuz Al Mamun, Kausar Parvin, Shirin Ziaei

**Affiliations:** 1 International Centre for Diarrhoeal Disease Research Bangladesh, Dhaka, Bangladesh; 2 Institute for Medical Information Processing, Biometry and Epidemiology (IBE), Faculty of Medicine, Ludwig Maximilian University of Munich, Munich, Germany; 3 Queensland University of Technology, Brisbane, Queensland, Australia; 4 Department of Food Studies, Nutrition and Dietetics, Uppsala Universitet, Uppsala, Sweden; 5 Department of Women's and Children’s Health; International Child Health and Nutrition, Uppsala University, Uppsala, Sweden

**Keywords:** Cohort study, Interdisciplinary Research, Global Health

## Abstract

**Introduction:**

Repeated exposure to domestic violence (DV) is common among women; however, little is known about how women’s experiences of DV change over time. This study explores transitions in women’s experience of physical DV over a 19-year period (2001–2020) and identifies risk and protective factors for such transitions in violence using data from the MINIMat cohort study in rural Bangladesh.

**Methods:**

Data on physical DV were collected using a modified Conflict Tactic Scale from a cohort of 1078 women, from Matlab, a rural subdistrict of Bangladesh, recruited during pregnancy and followed up 10 and 18 years after the birth of the index child. Discrete-time Markov Chain and covariate-dependent Markov models were used to identify transitions across time points and associated and risk and protective factors among women who transitioned from: (1) no lifetime violence at enrolment to victimisation at 10-year follow-up and (2) lifetime victimisation at enrolment to revictimisation at 10-year follow-up. Risk and protective factors for revictimisation at 18-year follow-up were also identified.

**Results:**

Most women reporting lifetime physical DV at enrolment experienced revictimisation at 10-year follow-up (70%), while 30% of women without prior experience of DV reported new victimisation. Revictimisation was lower at 18-year follow-up among women victimised at both prior points (30%). Low agency in decision-making and high agency in mobility increased likelihood of physical DV at 10-year follow-up regardless of violence status at enrolment. Living with in-laws protected against revictimisation at 10-year follow-up (adjusted OR (aOR) 2.53; 95% CI 1.30 to 4.91). Higher age (aOR 0.87; 95% CI 0.80 to 0.95) and non-governmental organisation (NGO) membership (aOR 0.23; 95% CI 0.09 to 0.55) reduced risk of revictimisation at 18-year follow-up.

**Conclusion:**

Findings highlight the complex nature of women’s empowerment and its evolving relationship with DV over time. Tailored approaches to empowerment and targeting older women in NGO programmes may offer long-term protection.

**WHAT IS ALREADY KNOWN ON THE TOPIC**

- Domestic violence against women is a pattern of behaviour with repeated exposure being common over time.
- Due to the limited number of longitudinal studies on domestic violence against women, little is known about the variation in the experience of violence in women’s lives over time and risk and protective factors in the transitions.

**WHAT THIS STUDY ADDS**

- The study showed that women’s experience of physical domestic violence transitions over 19 years and the experience of physical domestic violence early in life was a predictor of experiencing this violence in the subsequent time points.
- Low decision-making and high mobility were the risk factors for the experience of physical domestic violence at earlier stages of women’s lives, while in the later stage, being older and having membership in a non-governmental Organisation (NGO) were protective factors against re-victimisation.

**HOW THIS STUDY MIGHT AFFECT RESEARCH, PRACTICE OR POLICY**

- These findings underscore the need for nuanced approaches to women’s empowerment in addressing domestic violence. While boosting decision-making power can protect younger women, increasing mobility without changing social norms may escalate violence. Targeting older women in NGO programmes could offer long-term protection.

## Introduction

 Violence against women (VAW) is a serious public health concern and a violation of human rights. It is widespread globally with one in three women ever experiencing intimate partner violence (IPV).^1^ A plethora of short and long-term physical and mental health consequences of VAW has been well documented.^2^,^4^ The social and economic costs of VAW, including IPV, happen to be enormous.^5^ Not surprisingly, elimination of all forms of VAW features as one of the sustainable development goals (SDGs).^6^

For programme and policy purposes, it is very important to identify the factors contributing to VAW.^7^ Most of the existing literature finds that women’s age^8 9^ and education^8 10 11^ are inversely associated with IPV, while poverty is positively associated with it.^812^,^14^ Findings on factors associated with IPV may vary from study to study due to methodological and contextual differences. Association between women’s income earning and IPV, for instance, may differ depending on contextual factors^14^ such as relative economic position of the partner and gender role expectations in the community.^15^

The nature of the association between participation in savings and credit and other programmes and IPV has often been debated, with some researchers claiming that it is positive,^16^,^19^ while some contend the opposite,^19^,^21^ and others show no association.^22^

Proponents of relative status theory or status inconsistency theory argue that consistent status between the intimate partners is associated with lower VAW,^23^ while the likelihood of VAW is higher in relationships characterised by differences in age, education, occupational prestige, etc .^24^ For example, research suggests that women with higher levels of education or earnings compared with their partners may experience an elevated risk of IPV, as these differences in status can challenge traditional gender roles and provoke backlash from men who perceive this status inconsistency as a threat to their authority.^25 26^ Analysis of the WHO multicountry study data, however, shows either a non-existent relationship or a weak one between age gap between partners and IPV in different countries.^8^

Literature from both high-income countries (HICs) and low- and middle-income countries (LMICs) shows that egalitarian couple relationships were associated with a lower likelihood of IPV.^27^,^32^

In patriarchal settings such as South Asia, women’s mobility is treated as an important indicator of women’s power.^22 33^ Unfortunately, no study has examined its association with IPV. Conflicting findings have been presented regarding the association between living with in-laws and IPV. While some researchers have reported that co-residence with in-laws is associated with an increased risk of IPV,^34^,^36^ others have found no significant association between the two.^10^ In patrilocal settings like Bangladesh, where women typically move into their husband’s home after marriage, living with in-laws may reinforce traditional gender norms and power hierarchies that limit women’s autonomy.^37^

Unfortunately, most of the literature on factors contributing to VAW is based on cross-sectional data, which does not allow drawing any causal inference. A systematic review and meta-analysis of prospective longitudinal studies on the risk and protective factors for experiencing IPV^38^ finds that older age protected women from experiencing IPV. The literature is, however, inconclusive about the protective effect of education on physical IPV. Poverty was identified as a risk factor for IPV in one study, while no effect of it was revealed in another one.

Another gap in the literature lies in the fact that while prevalence of VAW happens to be relatively higher in LMICs,^1 38^ most of the studies on risk and protective factors for VAW come from HICs. The study by Yakubovich *et al* reveals that 55 out of 60 studies came from HICs.^38^ It is clear that in order to achieve VAW-related SDGs, it is critical to conduct more longitudinal studies in LMICs so that the risk and protective factors could be identified, opening opportunities for developing better programmes and policies.

Experiences of violence among women, however, may vary over time depending on different factors such as life stage, individual, family and community characteristics. It is imperative to better understand the transitions in VAW over time for the purpose of better targeting and for better informing programmes and policies.

To address these gaps in the literature, this paper examines transitions in married women’s experience of physical domestic violence (DV) perpetrated by the husband and in-laws over the course of a 19-year period in a rural area of Bangladesh and identifies the associated risk and protective factors of this violence experienced by women going through different transitions.

### The context

Bangladesh reports one of the highest rates of IPV in the world with 50% of ever-married women reporting lifetime physical IPV and 21% reporting such violence during the last 12 months.^39^ High rates of IPV in Bangladesh are in line with its patriarchal social structure,^40^ where VAW is highly condoned.^41^

In rural Bangladesh, 29% of the married women live with their in-laws (Bangladesh Demographic and Health Survey 2017–2018 unpublished data). In-laws are also reported to perpetrate VAW.^42^ Even when rural women live in nuclear families, they usually share the same compound with their in-laws.^43^ Women’s mobility is restricted,^44^ and most of them are not allowed to earn an income,^45^,^47^ and their decision-making power is usually limited.^46^ Low status, lack of access to resources and little power commanded by women in the marital home make them vulnerable to abuse not only by the husband but also by the in-laws.

## Methods

### Study design and setting

This study was part of the larger Maternal and Infant Nutrition Interventions in Matlab (MINIMat) trial (reg#ISRCTN16581394). MINIMat is a population-based food and micronutrient supplementation longitudinal cohort trial for pregnant women. MINIMat launched in 2001 in Matlab, a rural subdistrict of Bangladesh located in the southeastern part of the country. Detailed information on the MINIMat trial is available elsewhere.^48 49^ Briefly, between November 2001 and October 2003, all pregnant women identified from icddr,b’s Health and Demographic Surveillance System in Matlab (n=4436) were invited to participate in the MINIMat trial. On receipt of consent, the pregnant women were randomised into two types of food and three types of micronutrient supplementation groups in a two-by-three factorial design. The women were then interviewed at their 30th week of gestation, at 10 years and 18 years after the birth of the index child to collect information on their experience of DV. In Bangladesh, childbirth outside marriage is rare and often unreported; therefore, all women in our study were married. The current analysis included 1078 women, for whom the information on physical DV was available in all three rounds.

### Data collection

Table 1 presents the details of outcome measures with duration of exposure at three data collection points. At all three rounds of data collection, information on socioeconomic and demographic characteristics and women’s decision-making autonomy, financial autonomy and mobility were collected from all women using standard pre-designed questionnaires. At enrolment (during 2001–2003), during their clinic visits at the 30th week of gestation, data were collected from 3504 pregnant women on their experience of lifetime physical DV perpetrated by the husband or in-laws (V_b_). The main reasons for loss to follow-up from the enrolment were: fetal loss (38%), women not located at the given address (11%), withdrawal from the study (14%), out-migration (21%), incomplete data (14%) and others (2%). At the 10-year follow-up (10 years after the birth of the index child, ie, during 2012–2013), a subset of 1356 women whose index child was born between 16 April 2002 and 11 June 2003 were further interviewed about their experience of DV from the birth of the index child up to 10-year follow-up (V_t_). At the 18-year follow-up (18 years after the birth of the index child, ie, during 2020–2021), the women successfully interviewed in the previous two rounds were approached for an interview, and 1126 of them were successfully interviewed about their experience of physical DV by their husband and/or a family member during the past 12 months (V_e_). The interviews were conducted through household visits at 10- and 18-year follow-ups. All the interviews were conducted face-to-face and in private by trained female interviewers.

**Table 1:** Measurement of women’s exposure to physical DV at different time points of data collection

Timing of data collection	Outcome measure	Notations for experience of physical DV*	State of transitions, notation	Notations for all possible transitions in physical DV
Enrolment	Experience of physical DV ever up to 30 weeks of pregnancy	$V_{b}=\left\{ \begin{array}{l} 1,\;if\;reported\;DV\;at\;Enrolment\;\\ 0,\;otherwise \end{array}\right.$		
10 years of index child	Experience of physical DV since birth of index child until 10-year follow-up	$V_{t}=\left\{ \begin{array}{l} 1,\;if\;reported\;DV\;at\;10-year\;follow-up\\ 0,\;otherwise \end{array}\right.$	Enrolment to 10 years, V_bt_	$V_{0t}=\left\{ \begin{array}{l} \begin{array}{c} V_{00},\;if\;V_{b}=0\;and\;V_{t}=0\;\\ V_{01},\;if\;V_{b}=0\;and\;V_{t}=1 \end{array} \end{array}\right.$
				$V_{1t}=\left\{ \begin{array}{l} \begin{array}{c} V_{10},\;if\;V_{b}=1\;and\;V_{t}=0\;\\ V_{11},\;if\;V_{b}=1\;and\;V_{t}=1 \end{array} \end{array}\right.$
18-year follow-up	Experience of physical DV in the past year at 18-year follow-up	$V_{e}=\left\{ \begin{array}{l} 1,\;if\;reported\;DV\;at\;18-year\;follow-up\\ 0,\;otherwise \end{array}\right.$	Enrolment to 10 years to 18 years, V_bte_	$V_{00e}=\left\{ \begin{array}{l} \begin{array}{c} V_{000},\;if\;V_{b}=0,\;V_{t}=0,\;V_{e}=0\;\\ V_{001},\;if\;V_{b}=0,\;V_{t}=0,\;V_{e}=1 \end{array} \end{array}\right.$
				$V_{01e}=\left\{ \begin{array}{l} \begin{array}{c} V_{010},\;if\;V_{b}=0,\;V_{t}=1,\;V_{e}=0\;\\ V_{011},\;if\;V_{b}=0,\;V_{t}=1,\;V_{e}=1 \end{array} \end{array}\right.$
				$V_{10e}=\left\{ \begin{array}{l} \begin{array}{c} V_{100},\;if\;V_{b}=1,\;V_{t}=0,\;V_{e}=0\;\\ V_{101},\;if\;V_{b}=1,\;V_{t}=0,\;V_{e}=1 \end{array} \end{array}\right.$
				$V_{11e}=\left\{ \begin{array}{l} \begin{array}{c} V_{110},\;if\;V_{b}=1,\;V_{t}=1,\;V_{e}=0\;\\ V_{111},\;if\;V_{b}=1,\;V_{t}=1,\;V_{e}=1 \end{array} \end{array}\right.$

* The abbreviation DV in the table refers to physical DV.

DV, domestic violence.

### Measurement

#### Outcome variable: physical DV

Women’s experience of physical DV was measured using a modified version of the conflict tactic scale.^50^ Six questions were asked with response options: yes/no. A typical example is, ‘has your husband or anyone else from his family ever slapped you or thrown something at you that could hurt you?’ (online supplemental table 1). A woman was considered exposed to any physical DV at a specific round of data collection if she responded ‘yes’ to any of the six items and coded ‘Yes=1’ and ‘No=0’ otherwise.

#### Covariates

Covariates to include in the study were selected based on previous literature.^810 51^,^57^ To ensure temporality, in each model, to the extent possible, we used covariates from the previous round. If any covariate was missing from the previous round, those were used from the same follow-up.

##### Demographic factors

Women’s age was taken from enrolment and 10-year follow-up. Their education was taken from an 18-year follow-up. Since females usually do not continue education post-marriage in rural Bangladesh,^58^ it is more or less time-invariant and thus applicable to all the time points. Age and education were recorded in years and used as continuous variables in the models.

##### Women’s agency

Women’s agency in decision-making was measured using seven items. Women were asked about participation in decision-making regarding small and large household purchases, what food should be given to children, how children should be disciplined, where and when to visit for her treatment and children’s treatments and visiting natal family (online supplemental table 1). A 3-point Likert scale was used to record the responses to each item: not at all or very little (0); to some extent (1); and to a large or full extent (2). A summative score was obtained (range: 0–14), with a higher score indicating higher decision-making autonomy. The summative score was then divided into low (if score≤7) and high (if score>7; reference) categories. Women’s mobility-related agency was measured based on her usual practice of going to a health centre or hospital: alone; with a young child; or with someone else (reference).

Women’s income earning status was categorised into yes, if she was earning an income during the survey (reference), and no, otherwise. Non-governmental organisation (NGO) membership was categorised into yes, if she was a member of any NGO, and no (reference), otherwise. NGOs included microcredit organisations and others.

##### Couple’s characteristics

Spousal age difference was categorised into <5 years (reference), 5–9 years and ≥10 years.

##### Household characteristics

Living arrangement was assessed by whether or not the woman lived with her in-laws. Living arrangements covered two categories: not living with in-laws (reference) and living with in-laws. The household wealth index was created using a principal component analysis of household assets. The factor scores were then divided into tertiles: low, medium and high (reference).

### Statistical analyses

Descriptive analyses were performed to describe the background characteristics of the study participants at enrolment. The main aim of this analysis was to explore the transitions in the experience of physical DV against women from enrolment to 10-year follow-up and subsequently from enrolment to 10 years to 18 years and to identify the risk and protective factors of violence during such transitions. We used the discrete-time Markov Chain (DTMC) to identify the transitions in women’s experience of physical DV at different stages of their lives. A Markov chain describes a sequence of possible events in which the probability of each event depends only on the state attained in the previous event,^59^ and a DTMC represents a random process that undergoes transitions from one state to another ‘state space’, where the ‘state space’ is a set of all possible states of a system.^59^ In our case, the outcome variable physical DV has two possible transition states—women’s experiences of violence and non-violence across three discrete time periods described in table 1. A total of six transitions in the experience of physical DV against women were identified: V_0t_, V_1t_, V_00e_, V_10e_, V_10e_ and V_11e_ (table 1). Due to the issue of non-convergence resulting from low cell frequencies during the regression analysis in three of the transitions, we could identify the risk and protective factors of the remaining three transitions in the experience of physical DV: V_0t_, V_1t_, V_11e_. We used covariate-dependent Markov models for binary outcomes, which identify associated factors for such transitions in time-dependent repeated measures data.^60^ The covariate-dependent Markov model can be used to quantify the contribution of other factors by fitting different logistic regressions for different transition types by considering transition probability as a dependent and other factors as independent variables.^61^ For the transition V,_11e_ we used all the covariates from the 10 year follow-up. However, data on agency in decision-making, mobility-related agency and NGO membership were missing at enrolment. Thus, we used these covariates from 10-year follow-up for transitions V_0t_ and V_1t_, whereas the other covariates were used from enrolment. The model estimates were further tested for goodness of fit. The likelihood ratio test was performed to compare the fitted models with the null model. All the analyses were performed using STATA V.15 (online supplemental document 1). The level of significance was set at 5%.

### Ethical considerations

The studies at enrolment and 10- and 18-year follow-ups obtained ethical approval from icddr,b’s Institutional Review Board (PR-2000–025, PR-12022 and PR-19101, respectively). The 18-year follow-up was additionally approved by the Swedish Research Ethics Authority (# 2021–00523). The research adheres to the WHO’s ethical and safety guidelines for investigating VAW.^62^ Informed consent was obtained from the participants every time they were interviewed. Interviews were conducted in private by trained gender-matched interviewers. The study participants were informed about the purpose and nature of the study, its expected benefits and risks and the voluntary nature of participation. At enrolment, women who reported experience of physical or sexual DV or suicidal ideation were offered mental health counselling.^63^ At the 10- and 18-year follow-ups, all the women interviewed were provided with information regarding services for women experiencing violence.

## Results

### Background characteristics

Table 2 presents the background characteristics of the study participants at enrolment. The mean age of women was 26 years, and the mean years of schooling was 5 years. They were much younger compared with their husbands, with almost half of the women (47%) being more than 10 years younger. About 88% of the women reported having a high level of decision-making ability. Agency in mobility for healthcare seeking was low, with only one-fifth of the women going to a health centre or hospital alone. Two-thirds of the women were NGO members. Only seven per cent of the women engaged in any income-generation. Two in three women (63%) lived with their in-laws. The background characteristics of women for different transitions (V_0t_, V_1t_, V_11e_) are presented in online supplemental table 2.

**Table 2:** Background characteristics of the study participants at enrolment, n=1078

Characteristic*	Full sample, 1078
Age	
Mean (SD, range)	26.40 (5.9, 14–44)
Education	
Mean (SD, range)	4.89 (3.7, 0–12)
Spousal age difference, %	
Less than 5 years	13.17 (142)
5–9	39.89 (430)
10 and above	46.94 (506)
Agency in decision-making, %	
Low	12.24 (132)
High	87.76 (946)
Mobility: usual practice of going to a health centre, %	
Alone	20.78 (224)
Yes, with children	67.81 (731)
Yes, with someone else	11.41 (123)
NGO membership, %	
Yes	66.88 (721)
Earn an income, %	
Yes	7.42 (80)
Living with in-laws	
Yes	62.51 (672)
Wealth index, %	
Low	36.36 (392)
Medium	34.23 (369)
High	29.41 (317)

* NGO participation, decision-making and mobility were missing at enrolment. These characteristics were reported from the 10-year follow-up.

NGO, non-governmental organisation.

### Transitions in physical DV against women over time

**Figure 1:**
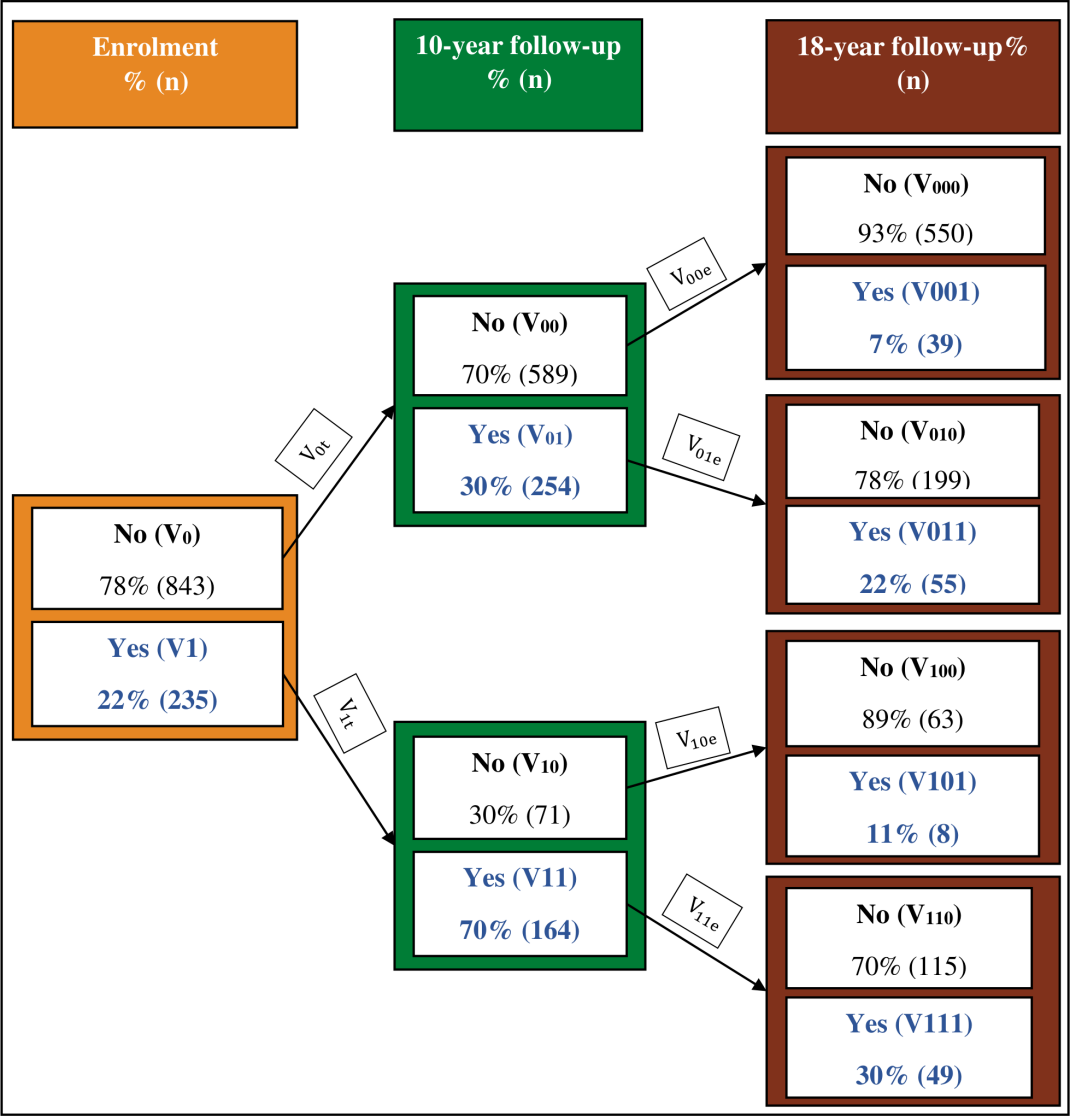
Tree Diagram of transition-specific prevalence in women’s experience of physical DV at different points in time.

Figure 1 presents the transition-specific prevalence in women’s experience of physical DV from one point in time to another: V_0t_, V_1t_, V_11e_. At enrolment, 22% of the women reported experiencing any lifetime physical DV up to 30 weeks of pregnancy (V_1_=22%). About 70% of the women who reported physical DV at enrolment also reported this violence at the 10-year follow-up (V_11_=70%), while 30% of the women who never reported this violence at enrolment reported it at the 10-year follow-up (V_01_=30%). Approximately 30% of the women who reported experiencing physical DV both at enrolment and 10 years also reported this violence at 18-year follow-up (V_111_=30%), while only seven per cent of the women who never reported this violence at any of the previous two time points reported it at 18-year follow-up (V_001_=7%). The prevalence of physical DV at 18-year follow-up among the women who had reported it at 10 years but not at enrolment was 22%, while it was 11% among those who had reported it at enrolment but not at 10-year follow-up.

The results clearly depict changes in women’s experience of physical DV at different points in life. The pattern of these changes shows that women who experienced violence at a particular stage were more vulnerable to experiencing violence at the next stage of life.

### Risk and protective factors for physical DV among women experiencing different transitions

Table 3 presents the risk and protective factors of women’s experience of physical DV in different transitions (V_01_, V_11_, V_111_) over a 19-year period. The identified factors in transitions V_01_ and V_11_ were completely different from the ones in transition V_111_. Low decision-making agency and high agency in mobility of women were found to be associated factors of physical DV for the transitions V_01_, and V_11_, but not for the transition V_111_. Women with lower decision-making agency were 1.7 times more likely to experience physical DV compared with their counterparts with higher decision-making agency for the transition V_01_ (adjusted OR (aOR) 1.65; 95% CI 1.02, 2.66) and 3.1 times for the transition V_11_ (aOR 3.05; 95% CI 1.25, 7.46). These results suggest that the association of physical DV was stronger in V_11_ compared with V_01_ among women with lower decision-making agency.

**Table 3:** Associated, risk and protective factors for physical domestic violence among women experiencing different transitions in violence from one time point to another (2001–2020)

Covariate	V_01_ aOR (95% CI)	V_11_ aOR (95% CI)	V _111_ aOR (95% CI)
Age	0.97 (0.95 to 1.01)	0.97 (0.92 to 1.04)	0.87 (0.80 to 0.95)*
Education	0.96 (0.92 to 1.01)	0.94 (0.84 to 1.06)	1.02 (0.88 to 1.18)
Spousal age difference			
< 5 years (ref)			
5–9	0.88 (0.55 to 1.40)	1.03 (0.41 to 2.58)	2.85 (0.77 to 10.58)
10 and above	0.75 (0.47 to 1.19)	1.46 (0.58 to 3.68)	0.73 (0.19 to 2.76)
Agency in decision-making			
High (ref)			
Low	1.65* (1.02 to 2.66)	3.05* (1.25 to 7.46)	0.97 (0.39 to 2.43)
Mobility: usual practice of going to a health centre or hospital			
With someone else (ref)			
Alone	2.11* (1.10 to 4.03)	3.04* (1.08 to 8.58)	1.08 (0.22 to 5.33)
With a child	2.29* (1.29 to 4.07)	2.97* (1.16 to 7.57)	0.88 (0.20 to 3.83)
NGO membership			
No (ref)			
Yes	1.27 (0.91 to 1.76)	0.88 (0.45 to 1.72)	0.23* (0.09 to 0.55)
Earn an income			
Yes (ref)			
No	1.39 (0.73 to 2.63)	1.17 (0.39 to 3.51)	5.08 (0.56 to 45.65)
Living with in-laws			
Yes (ref)			
No	1.23 (0.87 to 1.73)	2.53* (1.30 to 4.91)	0.97 (0.43 to 2.22)
Wealth index			
High (ref)			
Medium	0.98 (0.66 to 1.45)	0.60 (0.23 to 1.54)	2.85 (0.70 to 11.65)
Low	1.29 (0.83 to 2.00)	0.83 (0.31 to 2.24)	3.67 (0.89 to 15.11)
Test for goodness of fit of the models			
Likelihood ratio	34.28	25.87	36.60
d.f.	9	9	9
p Value	0.0006	0.01	0.0003

* *p*<0.05.

aOR, adjusted OR; NGO, non-governmental organisation.

High agency in mobility increased the odds of physical DV in the transitions V_01_ and V_11_; however, it had no effect on physical DV in the transition V_111_. For the transition V_01_, women who could go to a health centre or hospital alone and with children had 2.1 and 2.3 times higher odds, respectively, of experiencing physical DV compared with women who go with someone else. The odds of experiencing such violence in the transition V_11_ were even higher (alone (aOR: 3.04; 95% CI 1.08 to 8.58) and with children (aOR: 2.97; 95% CI 1.16 to 7.57)).

The women who did not live with their in-laws had 2.5 times higher odds of experiencing physical DV compared with those who did in the transition V_11_ (aOR 2.53; 95% CI 1.30 to 4.91). Higher age and NGO membership played a protective role against physical DV in the transition V_111,_ but not in the transitions V_01_, and V_11_. In the transition V_111_, a one-unit increase in the age of a woman decreased the likelihood of physical DV by 13% (aOR 0.87; 95% CI 0.80 to 0.95), while the women who were NGO members had 77% lower odds of experiencing physical DV compared with the women who were not members of NGOs (aOR: 0.23; 95% CI 0.09 to 0.55).

The model estimates presented were further tested for goodness of fit of the models. The likelihood ratio test indicates that all three models V01, V11 and V111 fit significantly better than the null model (see p values in table 3).

## Discussion

This study shows that women’s experience of physical DV underwent different transitions over a period of 19 years. Experience of physical DV early in life was a predictor of this violence in the subsequent time points. Thus, once a violent dynamic was established early in a relationship, it held for at least 10 years for most of the women. This finding lends support to existing literature regarding early onset of violence.^1^ It is also in line with the claim that VAW is a pattern of behaviour and therefore violence in a family is sustained over a relatively long period.^64 65^

Cross-sectional studies also suggest that the prevalence of VAW increases with age until it reaches its peak when a woman is aged between 30 and 39 years and then it declines.^1^ Our data also show a similar pattern. For some women, the onset of physical DV may, however, occur at a later time point (10-year follow-up). Most of the women who experienced a later onset (V_01_), did not, however, experience physical DV in the subsequent period (V_010_=78%).

Our analyses of transition-specific associated, risk and protective factors for physical DV among women show that they were not identical across transitions. While the factors associated with transitions from no victimisation to onset of violence at 10-year follow-up and from victimisation at enrolment to re-victimisation at 10-year follow-up shared greater commonality than differences, they were in stark contrast to the risk and protective factors revealed in transition from victimisation at the first two time points to re-victimisation at the third.

Two important dimensions of women’s agency, namely, women’s decision-making power and mobility, were associated with physical DV in transitions V_01_ and V_11_. Women’s low decision-making agency increased the odds of experiencing physical DV compared with women with high decision-making agency for the transitions V_01_ and V_11_. For the same transitions, high agency in mobility increased the odds of physical DV. These findings echo the finding by Ma *et al* based on an analysis of nationally representative data from 53 LMICs.^32^ Our finding further shows that the nature of the relationship between women’s agency and VAW may be nuanced rather than unidirectional. Thus, in our social context, while low decision-making power was associated with a higher likelihood of DV, higher agency in mobility increased the likelihood of physical DV in the transitions V_01_ and V_11_. We contend that low decision-making power indicates low status in a household and makes women vulnerable to this violence. On the other hand, mobility without a chaperon, an indicator of higher agency, actually increased the likelihood of physical DV in both these transitions. This may be due to a strong impetus to control younger women’s sexuality and consequently their mobility, which heightens the risk of physical DV of a woman who goes to places without a chaperon. The fact that such mobility did not come out as a risk factor for violence in V_111_ may be explained by abated concerns regarding women’s sexuality and mobility when they are much older.

Two decades ago, in a study of two different districts of Bangladesh, Koenig *et al*^66^ showed that the direction of association between women’s agency and IPV may be different depending on contextual factors. Thus, in one of the districts, where women’s agency was relatively higher compared with the other district, the higher agency of an individual woman was not associated with IPV. In contrast, in the other district, where women’s agency was relatively low, an individual woman’s higher agency increased her likelihood of experiencing IPV. Our findings show that within the same sub-district, different dimensions of women’s agency may have different types of association (ie, positive or negative) with DV at different stages of the life of a woman. Thus, a methodological point to note is that greater insights can be achieved by separating these different dimensions of agency in the analysis.

These dimensions of women’s agency in transitions V_01_ and V_11_, however, did not have any effect on violence in transition V_111_. This shows that the impact of women’s agency on violence may also depend on women’s life stage. The absence of any effect of mobility on DV in transition V_111_ may be explained by differences in social norms depending on the life stage a woman is in. As mentioned above, younger women are expected to have more stringent restrictions on their mobility^67^ compared with older women. However, it is not clear to us why decision-making power did not provide protection from DV in transition V_111_.

While NGO membership had no association with physical DV among women in the transitions V_01_ and V_11_, it provided protection against the subsequent transition V_111_. These findings for transitions V_01_ and V_11_ in women’s experience of violence need to be considered in light of the existing literature that shows no effect of NGO membership on IPV in a sample of women from Matlab.^22^ A protective effect of NGO membership among women experiencing physical DV in the transition V_111_ may be explained by older age of women in this group compared with those who experienced physical DV in transitions V_01_ and V_11_ (26–28 vs 39 years). NGO programmes in Bangladesh target economically disadvantaged women. While access to financial resources is likely to be appreciated in this resource-poor context, participation in group sessions may compete with younger women’s childcare responsibilities and household chores accruing them no protection against physical DV. Absence of these constraints at an older age may have enabled women with experience of physical DV in the transition V_111_ to gain this protection. Thus, our findings reveal that unpacking the effect of NGO membership by different age groups of women may actually tell us a different story. These findings suggest that different gender role expectations at different life stages may underlie differences in how a factor may contribute or not contribute to physical DV against women.

In many contexts, the isolation of women and living in a nuclear family is known to increase their vulnerability to violence.^68^,^70^ Although our finding is in line with this literature, it is in conflict with a strand of literature from Bangladesh. In Bangladesh, marriages are patrilocal. On marriage, a bride usually joins an extended marital family headed by the father-in-law, where the mother-in-law is usually in charge of running the household. Qualitative studies show that conflict with a mother-in-law is a huge issue in an extended family. Mothers-in-law are commonly known to instigate VAW in Bangladesh.^54 71^ In general, a woman tends to prefer to live in a nuclear family, where she is more in control. Formation of a nuclear family usually occurs later in marriage. Thus, a negative association between living with in-laws and physical DV at V_11_ seems counter-intuitive. However, our finding is in line with quantitative findings from India and Bangladesh.^66 72^ In a study conducted in two rural districts of Bangladesh differing substantially in cultural conservativeness, Koenig *et al*^66^ found that extended family is negatively related to a woman’s experience of physical violence both in pooled and site-wise analyses. They explained this result by the suppression of physical VAW by the mother-in-law. A similar argument was put forward by Clark *et al*^73^ in explaining a similar finding from Nepal. More research needs to be conducted to better understand contradictory results from qualitative and quantitative findings from the same context.

While women’s higher education, higher spousal age difference and women’s income earning were found to be associated with IPV in other studies,^810 14 15 74^,^76^ our study did not find any causal relationship between these factors and DV.

Some limitations of this study deserve to be noted. This study was conducted in Matlab, Bangladesh, making the findings not generalisable more widely. Still, unlike many non-population-based studies, coverage of all pregnant women at enrolment and follow-ups covering all women giving birth during a specific window of time allowed for inferences to a defined population.

VAW is almost always under-reported. It is true that the level of physical DV reported by our study participants was lower than the level of spousal physical violence reported by reproductive-aged women in Matlab^77^ at enrolment and in the 18-year follow-up. However, these rates are not comparable due to differences in the study populations (eg, age). It is noteworthy that we have used standard tools, followed WHO ethical and safety guidelines regarding surveys on VAW,^62^ and interviewed in a private place and non-judgementally to enhance disclosure.

The reference periods for these three time points were different. Thus, the prevalence rates could not be compared with each other. The age of all the study participants was not the same. So, the life stages that we are referring to may be somewhat diffused. However, all the women were undoubtedly younger at recruitment compared with the 10- and 18-year follow-up periods. Most women in Bangladesh complete family at a relatively young age (by 27 years on average).^78^ Since the women were pregnant at recruitment, they were thus young reproductive-aged women at recruitment. In each of the models, we have controlled for age to identify the risk and protective factors for physical DV.

Data for important factors such as agency in decision-making, mobility-related agency and NGO membership were not available at enrolment. Thus, we had to use these variables from the 10-year follow-up, which did not allow us to maintain temporal ordering between the input and output variables for transitions V_01_ and V_11_. However, we could ensure temporality for the transition V_111_. Due to non-convergence, we could not identify the risk and protective factors for physical DV in all the transitions observed in the data. Due to the small number of participants from each studied village, we could not identify community-level risk and protective factors for physical DV. And finally, our study collected data on DV by any member of the husband’s family, including the husband. As a result, it was not possible to distinguish risk and protective factors for husband-versus-in-law-perpetrated violence.

We would like to highlight the following strengths of this study. First, the analysis was based on longitudinal prospective data on a cohort of women enabling us to make causal inferences. Second, repeated measurement of violence in the cohort allowed us to examine the pattern of physical DV over time. Third, the nature of the data also allowed us to identify the transitions in this violence from one point in time to another and to investigate associated factors for V_01_ and V_11_ and the risk and protective factors for this violence for transition V_111_.

This study makes a major contribution to the literature by expanding our understanding of the nuanced association of different dimensions of women’s agency on violence in different time points and underlines the importance of analysing different dimensions of agency separately for greater insights and for programme and policy purposes. Moreover, to our knowledge, no study to date has shed light on the risk and protective factors for VAW exposed to physical DV for three consecutive periods spanning over a 19-year period.

These findings have major implications for research, programmes and policies. Our findings show that it is important to better understand the context and the dimensions of women’s empowerment that may have a nuanced association with physical DV. They also suggest that increasing women’s decision-making power may have a beneficial effect on relatively young women. But increasing women’s agency in mobility will have to go hand in hand with social norm change around control over women’s sexuality, purdah and restricted mobility of women. Increasing women’s agency in mobility without creating an enabling or supporting environment may escalate this violence. Targeting older women in NGO programmes would offer women victimised over a long period of time (ie, at least over 10 years) protection from being further victimised. More longitudinal studies need to be undertaken for a better understanding of the other transitions in physical DV, transitions occurring in other types of DV and their multilevel risk and protective factors.

## Supplementary material

10.1136/bmjgh-2024-018458 online supplemental file 1

10.1136/bmjgh-2024-018458 online supplemental table 1

10.1136/bmjgh-2024-018458 online supplemental table 2

## Data Availability

Data are available upon reasonable request.
