# Supplementary material for: Transitions in women’s experience of physical domestic violence during 2001–2020 and related risk and protective factors: the MINIMat longitudinal cohort study in rural Bangladesh
Source: BMJ Glob Health. 2025 Dec 23;10(12):e018458. doi: 10.1136/bmjgh-2024-018458 (PMC12730756; doi:10.1136/bmjgh-2024-018458)
Supplement: online supplemental file 1 [file bmjgh-10-12-s001.docx]

**Supplementary document _STATA syntax:**

***Create outcome variables**

gen pv_01 = any_pv_ever_preg

gen pv_001 = .

replace pv_001 = 1 if pv_01 == 0 & any_pv_after_birth ==1

replace pv_001 = 0 if pv_01 == 0 & any_pv_after_birth ==0

lab var pv_001 “physical violence in transition 0-1”

label define pv_001 0 "No" 1 "Yes"

gen pv_011 = .

replace pv_011 = 1 if pv_01 == 1 & any_pv_after_birth ==1

replace pv_011 = 0 if pv_01 == 1 & any_pv_after_birth ==0

lab var pv_011 “physical violence in transition 1-1”

label define pv_011 0 "No" 1 "Yes"

gen pv_0111 = .

replace pv_0111 = 1 if pv_011 == 1 & any_pv_12months_18 ==1

replace pv_0111 = 0 if pv_011 == 1 & any_pv_12months_18 ==0

lab var pv_0111 “physical violence in transition 1-1-1”

label define pv_0111 0 "No" 1 "Yes"

***Determinants of transitions- Logistic regression for discrete type Markov Chain**

logit pv_001 age_preg q102_18 i.age_diff_spouse_cat ib1.family_structure_0 ib3.wealth_index_0_3 ib1.earn_income_0 ib1.decision_scr_cat_10 i.NGO_participation_10_yn ib3.q402_a_10, or

logit pv_0111 age_10Y q102_18 i.age_diff_spouse_cat ib1.family_structure_10 ib3.wealth_index_10_3 ib1.earn_income_10 ib1.decision_scr_cat_10 i.NGO_participation_10_yn ib3.q402_a_10 , or

logit pv_0111 age_10Y q102_18 i.age_diff_spouse_cat ib1.family_structure_10 ib3.wealth_index_10_3 ib1.earn_income_10 ib1.decision_scr_cat_10 i.NGO_participation_10_yn ib3.q402_a_10 , or

***Data labeling**

lab var age_preg “Age at enrolment”

lab var q102_18 “Education level”

lab var age_diff_spouse_cat_10 “Age difference with husband”

label define age_diff_spouse_cat_10 1 "< 5" 2 "5-9" 3 "10 and above"

lab var family_structure_0 “Living with in-laws”

label define family_structure_0 0 "No" 1 "Yes"

lab var wealth_index_0_3 “Wealth index”

label define wealth_index_0_3 1 "Low" 2 "Medium" 3 “High”

lab var earn_income_0 “Earn an income”

label define earn_income_0 0 "No" 1 "Yes"

lab var decision_scr_cat_10 "Decision making ability"

label define decision_scr_cat_10 0 "Low decision making ability" 1 "High decision making ability"

lab var NGO_participation_10_yn “NGO participation”

label define NGO_participation_10_yn 0 "No" 1 "Yes"

lab var q402_a_10 “Practice going to health centre”

label define q402_a_10 1 "Alone" 2 "with a child" 3 “with someone else”
