# Supplementary material for: Transitions in women’s experience of physical domestic violence during 2001–2020 and related risk and protective factors: the MINIMat longitudinal cohort study in rural Bangladesh
Source: BMJ Glob Health. 2025 Dec 23;10(12):e018458. doi: 10.1136/bmjgh-2024-018458 (PMC12730756; doi:10.1136/bmjgh-2024-018458)
Supplement: online supplemental table 1 [file bmjgh-10-12-s002.docx]

**Supplementary Table 1. Items used to measure physical violence and women’s agency in decision-making**

| **Question** | **Time point used in the analysis** | | |
| --- | --- | --- | --- |
|  | Enrolment | 10-year follow-up | 18-year follow-up |
| **Physical DV^1^** | | | |
| Has your husband or anyone else from your family ever - |  |  |  |
| 1. slapped you or threw something at you that could hurt you? | √ | √ | √ |
| 1. pushed you or shoved you? |  |  |  |
| 1. hit you with his fist or with something else that could hurt you? |  |  |  |
| 1. kicked you, dragged you or beat you up? |  |  |  |
| 1. choked or burnt you on purpose? |  |  |  |
| 1. used a knife, gun or other weapon against you? |  |  |  |
| **Women’s agency in decision-making** |  |  |  |
| 1. How much can you decide/authority on making smaller household purchases for daily household needs (salt, oil, kerosene, cloths)? |  | √ |  |
| 1. How much can you decide on making larger household purchase (such as land, goat, cattle, boat, motorcycle etc.)? |  |  |  |
| 1. How much can you decide what food should be given to the children? |  |  |  |
| 1. How much can you decide how your children should be disciplined |  |  |  |
| 1. How much can you decide when and where you should take a sick child or treatment |  |  |  |
| 1. How much can you decide when and where you go when you fall sick |  |  |  |
| 1. How much can you decide to go and visit your family/relatives who are living elsewhere |  |  |  |

^1^ Response options: ‘Yes = 1’, and ‘No = 0’ otherwise.
