# Supplementary material for: Transitions in women’s experience of physical domestic violence during 2001–2020 and related risk and protective factors: the MINIMat longitudinal cohort study in rural Bangladesh
Source: BMJ Glob Health. 2025 Dec 23;10(12):e018458. doi: 10.1136/bmjgh-2024-018458 (PMC12730756; doi:10.1136/bmjgh-2024-018458)
Supplement: online supplemental table 2 [file bmjgh-10-12-s003.docx]

**Supplementary Table 2. Demographic characteristics of study participants by transitions, n=1,078**

| **Characteristic** | **V_01_, 843** | **V_11_, 235** | **V_111_, 164** |
| --- | --- | --- | --- |
| *Covariate used from* | *Enrolment* | *Enrolment* | *10Y* |
| **Age, mean (SD, range)** | 25.90 (6, 14-43) | 28.20 (5.37, 17-44) | 39.06 (5.26, 28-53) |
| *Covariate used from* | *18Y* | *18Y* | *18Y* |
| **Education, mean (SD, range)** | 5.30 (3.71, 0-12) | 3.4 (3.27, 0-12) | 3.08 (3, 0-12) |
| *Covariate used from* | *Enrolment* | *Enrolment* | *10Y* |
| **Spousal age difference, %** |  |  |  |
| < 5 years | 13.05 (110) | 13.62 (32) | 12.80 (21) |
| 5-9 | 39.03 (329) | 42.98 (101) | 41.46 (68) |
| 10 and above | 47.92 (404) | 43.40 (102) | 45.73 (75) |
| *Covariate used from* | *10Y* | *10Y* | *10Y* |
| **Agency in decision-making, %** |  |  |  |
| Low | 10.08 (85) | 20.00 (47) | 23.78 (39) |
| High | 89.92 (758) | 80.00 (188) | 76.22 (125) |
| **Mobility: usual practice of going to a health centre, %** |  |  |  |
| Alone | 19.34 (163) | 25.96 (61) | 26.22 (43) |
| Yes, with children | 69.16 (583) | 62.98 (148) | 65.85 (108) |
| Yes, with someone else | 11.51 (97) | 11.06 (26) | 7.93 (13) |
| **NGO participation, %** |  |  |  |
| Yes | 66.19 (558) | 69.36 (163) | 67.68 (111) |
| *Covariate used from* | *Enrolment* | *Enrolment* | *10Y* |
| **Earn an income, %** |  |  |  |
| Yes | 7.00 (59) | 8.94 (21) | 7.93 (13) |
| **Living with in-laws, %** |  |  |  |
| Yes | 65.04(548) | 53.42(125) | 31.10 (51) |
| **Wealth index, %** |  |  |  |
| Low | 39.03 (329) | 59.57 (140) | 53.05 (87) |
| Medium | 20.88 (176) | 21.70 (51) | 23.17 (38) |
| High | 40.09 (338) | 18.72 (44) | 23.78 (39) |
